# Comparative Analysis of Disease-Linked Single Nucleotide Polymorphic Markers from *Brassica rapa* for Their Applicability to *Brassica oleracea*

**DOI:** 10.1371/journal.pone.0120163

**Published:** 2015-03-19

**Authors:** Young-Il Cho, Yul-Kyun Ahn, Swati Tripathi, Jeong-Ho Kim, Hye-Eun Lee, Do-Sun Kim

**Affiliations:** Vegetable Research Division, National Institute of Horticultural & Herbal Science, Rural Development Administration, Suwon, Republic of Korea; New Mexico State University, UNITED STATES

## Abstract

Numerous studies using single nucleotide polymorphisms (SNPs) have been conducted in humans, and other animals, and in major crops, including rice, soybean, and Chinese cabbage. However, the number of SNP studies in cabbage is limited. In this present study, we evaluated whether 7,645 SNPs previously identified as molecular markers linked to disease resistance in the *Brassica rapa* genome could be applied to *B*. *oleracea*. In a BLAST analysis using the SNP sequences of *B*. *rapa* and *B*. *oleracea* genomic sequence data registered in the NCBI database, 256 genes for which SNPs had been identified in *B*. *rapa* were found in *B*. *oleracea*. These genes were classified into three functional groups: molecular function (64 genes), biological process (96 genes), and cellular component (96 genes). A total of 693 SNP markers, including 145 SNP markers [BRH—developed from the *B*. *rapa* genome for high-resolution melt (HRM) analysis], 425 SNP markers (BRP—based on the *B*. *rapa* genome that could be applied to *B*. *oleracea*), and 123 new SNP markers (BRS—derived from BRP and designed for HRM analysis), were investigated for their ability to amplify sequences from cabbage genomic DNA. In total, 425 of the SNP markers (BRP-based on *B*. *rapa* genome), selected from 7,645 SNPs, were successfully applied to *B*. *oleracea*. Using PCR, 108 of 145 BRH (74.5%), 415 of 425 BRP (97.6%), and 118 of 123 BRS (95.9%) showed amplification, suggesting that it is possible to apply SNP markers developed based on the *B*. *rapa* genome to *B*. *oleracea*. These results provide valuable information that can be utilized in cabbage genetics and breeding programs using molecular markers derived from other *Brassica* species.

## Introduction

The genus *Brassica* is one of the most important vegetable crop genera in the world. *Brassica* crops provide vegetables, oil, fodder, and condiments and are also valuable sources of dietary fiber, vitamin C, and other beneficial factors, including several anticancer compounds [1,2]. In addition, *Brassica* species are popular for producing high-quality biodiesel owing to their relatively low levels of polyunsaturated and saturated fatty acids [3].

Among *Brassica* species, *Brassica rapa* (AA, 2n = 20), *Brassica nigra* (BB, 2n = 16), and *Brassica oleracea* (CC, 2n = 18) are diploid, whereas *Brassica juncea* (AABB, 2n = 36), *Brassica napus* (AACC, 2n = 38), and *Brassica carinata* (BBCC, 2n = 34) are amphidiploid (i.e., having combinations of the genomes of these diploid species) [4]. Thus, the *Brassica* genome provides substantial opportunities for studying the divergence of gene function and genome evolution associated with polyploidy, extensive duplication, and hybridization [5]. *Brassica rapa* has a small genome (529 Mb) compared with its close diploid relatives *B*. *oleracea* (696 Mb) and *B*. *nigra* (632 Mb) [6,7]. These characteristics are useful for the study of genomic traits. In response to the need for a simple genetic system with favorable genetic attributes for research on *Brassica* species, *B*. *rapa* has become a model species representing the *Brassica* A genome and is the focus of multifaceted genome projects with the goal of whole-genome sequencing based on the clone-by-clone strategy (http://www.brassica.info) [8].

Single nucleotide polymorphisms (SNPs) are the most common type of variation in DNA [9]. A SNP is a unique nucleotide difference between two DNA sequences. In theory, SNP variations could involve four different nucleotides at a particular site, but actually only two of these four possibilities are usually observed. Thus, in practice, SNPs are biallelic markers, and therefore the information content of a single SNP is limited compared to polyallelic simple sequence repeat (SSR) markers [10,11]. This disadvantage is overcome by the relatively greater abundance and stability of SNP loci compared to SSR loci. The abundance, ubiquity, and interspersed nature of SNPs together with the potential for automatic high-throughput analysis make them ideal candidate molecular markers for the construction of high-density genetic maps, quantitative trait loci (QTL) fine mapping, marker-assisted plant breeding, and genetic association studies [12,13]. In addition, SNPs located in known genes provide a fast alternative to analyzing the fate of agronomically important alleles in breeding populations, thus providing functional markers [14]. SNPs may be used as simple genetic markers, which may be identified in the vicinity of virtually every gene [13]. There is also great potential for the use of SNPs in the detection of associations between allelic forms of a gene and phenotypes, especially for common diseases with multifactorial genetics [15]. SNP discovery has been reported for several plant species, and the frequency of SNPs has shown variation depending on the different genomic regions in plants [8].

Genomes sequencing projects for *Brassica* species, including *B*. *rapa*, have produced vast amounts of sequence data that will provide useful information for genetic studies [3,16–18]. In total, 21,311 SNPs and 6,753 InDels in the gene space of the *B*. *rapa* genome were identified by re-sequencing 1,398 sequence-tagged sites (STSs) in eight genotypes [8]. In addition, more than 37,000 SNPs were identified through a comparison of two accessions of the model plant *Arabidosis thaliana* [19]. Cavell et al. [20] reported that the close sequence identity of coding regions (~87%) between the genomes of *Brassica* species and *A*. *thaliana* would allow for detailed comparative analyses. Such comparative mapping studies [21–23] have allowed for the assignment of orthologous segments in *Brassica* species and *A*. *thaliana*, enabling the identification of candidate genes that may directly account for *Brassica* QTL. These informational and genomic resources will promote the genome-wide study of DNA polymorphisms in *B*. *rapa* and will contribute significantly to *Brassica* crop improvement. Furthermore, the availability of *B*. *rapa* genomic sequence data offers an unprecedented opportunity to conduct detailed comparative analysis of the relationships between *Brassica* species genomes, and between the *Brassica* genome and *A*. *thaliana* genome. Various DNA markers, including AFLP [24], PCR-based markers [25,26], RFLP [27], and SSRs [28,29], have been studied in *B*. *oleracea*. However, there is no information available regarding the comparative marker profile of *B*. *rapa* and *B*. *oleracea* genome.

In the present study, we evaluated whether 7,645 SNPs linked to disease resistance from the *B*. *rapa* genome may be applied to *B*. *oleracea*. A total of 693 SNP markers, including 145 SNP markers (BRH) developed from the *B*. *rapa* genome for high-resolution melt (HRM), 425 SNP markers (BRP) based on the *B*. *rapa* genome that could be applied to *B*. *oleracea*, and 123 new SNP markers (BRS) derived from BRP and designed for HRM analysis, were found to be useful tools for QTL fine mapping, the development of SNP markers linked to disease resistance, genomics-based breeding, and genetic association studies in *B*. *oleracea*.

## Materials and Methods

### Plant materials and DNA extraction

To evaluate the utility of SNP markers based on the *B*. *rapa* genome for *B*. *oleracea*, two cabbage varieties, Chungam45 and Bogam3, were selected from 53 cabbage accessions. Plants from each variety were container-grown in a greenhouse at the National Institute of Horticultural and Herbal Science of the Rural Development Administration (RDA). DNA was extracted from fresh, young leaves of two plants using a DNA extraction kit (Qiagen, Hilden, Germany). The relative purity and concentration of the extracted DNA were estimated with ND-1000 spectrophotometer (NanoDrop Technologies, Inc., Wilmington, DE, USA), and the final concentration of each DNA sample was adjusted to 20 ng/μL.

### Functional analysis

Previously, we developed 21,311 SNPs and 6,753 InDels using the gene space of the *B*. *rapa* genome by re-sequencing 1,398 STSs in eight genotypes [8]. The sequences of 7,645 of 21,311 SNP markers, which were linked to disease resistance based on the *B*. *rapa* genome and which aligned to BAC sequences of *B*. *rapa*, were obtained using FGENESH (http://www.softberry.com) based on a *B*. *rapa* matrix, in order to confirm the positions of the SNP primers and to analyze the information according to the positions of the corresponding genes. To analyze the biological functions of the predicted genes, the protein sequences of the corresponding genes were extracted from the BAC sequences collected in the *B*. *rapa* Genome Project (http://www.brassica-rapa.org/BRGP/chromosomeSequence.jsp). hese sequences were analyzed for function using the UniProt database (Table 1). A functional analysis of each protein was conducted according to its characteristics using MIPS, FunCat, Gene Ontology (GO), and Clusters of Orthologous Groups. The unigenes of 7,645 SNP markers, which were designed from the *B*. *rapa* genome, were mapped to the *B*. *oleracea* and *A*. *thaliana* genomes using the BLAST (version 2.2.24) program with an e-value of 1e-4 (top match: 1).

**Table 1:** Databases used to compare 7,645 SNP markers developed from the *Brassica rapa* genome with the *Brassica oleracea* genome.

Analysis	Content	URL
Blocks	Multiply aligned ungaped segments corresponding to the most highly conserved regions of proteins	http://blocks.fhcrc.org
COG	Clusters of orthologous groups	http://www.ncbi.nlm.nih.gov/COG
EMBL	First DNA database in Europe	http://www.ebi.ac.uk/embl
FunCat	Functional catalog from MIPS	http://mips.helmholtz-muenchen.de/proj/funcatDB
PDB	Database of protein and nucleic acid three dimensional structure	http://www.rcsb.rog/pdb/home/home.do
Pfam	Protein domain database from the Sanger Center	http://www.sanger.ac.uk/Software/Pfam
PROSITE	Databases from the Swiss Institute of Bioinformatics	http://www.expasy.ch/prosite
SCOP	Structural classification of proteins	http://scop.mrc-lmb.cam.ac.uk/scop
UniProt	Universal Protein Resource(SWISS-PROT and TrEMBL)	http://www.ebi.uniprot.org/index.html
GO	Gene Ontology	http://www.geneontology.org

### Primer design and PCR

The selected 7,645 SNP markers were previously designed from flanking exon sequences of the selected genes to amplify genic regions, including introns, by means of the Primer3 program [8,30]. In total, 693 SNP primers were developed and used in this study. Of these, 145 BRH primers were newly developed for HRM analysis using Primer3, and 425 BRP primers that could be applied to *B*. *oleracea* were selected from the 7,645 SNP primers. A total of 123 BRS primers were newly designed using CLC Genomic Workbench (CLC bio, Aarhus, Denmark) and Primer3. The amplification reactions were carried out in a total volume of 20 μl containing 40 ng of genomic DNA as template, 0.5 μM forward and reverse primers, and 2 × GoTaq Green Master Mix (Promega, Madison, WI, USA) following the manufacturer’s recommended protocols. PCR was conducted as follows: 95°C for 2 min, followed by 35 cycles of 95°C for 30 s; 55, 57, 60, 62, or 65°C for 30 s; and 72°C for 1 min, with a final extension at 72°C for 10 min using an Eppendorf Thermocycler (Eppendorf, Germany). Electrophoresis on a 1.0% agarose gel with ethidium bromide confirmed the presence of the amplified products.

## Results and Discussion

### Comparison of the *B*. *rapa* SNP primer sequences and with the *B*. *oleracea* genome

To evaluate the applicability of SNP markers designed from the *B*. *rapa* genome to *B*. *oleracea*, all reference sequences of *B*. *oleracea* (742,612), including mRNAs (1,772), ESTs (59,946), and genome survey sequences (GSSs) (680,894), were assembled from the NCBI database (http://www.ncbi.nlm.nih.gov/) (Table 2). The protein (1,735) and final protein sequences (nrProtein) (20,632) of *B*. *oleracea* as a non-redundant protein dataset were also assembled for ortholog analysis. In addition, 194,305 ESTs, 198,585 GSSs, 3,965 mRNAs, 2,062 proteins, and 55,420 nrProtein sequences of *B*. *rapa* were collected from the NCBI database. The close phylogenetic relationship between the *Brassica* species and the model plant *A*. *thaliana* suggests that the transfer of knowledge from *Arabidopsis* for *Brassica* crop improvement would be straightforward [5]. Extensive gene loss or gain events and large-scale chromosomal rearrangements, including segmental duplications or deletions, in the *Brassica* lineage complicated the orthologous relationships between loci from the two genomes [31]. Hybridization between species is another source of *Brassica* genome complexity. The complex genomic organization of *Brassica* species as a result of multiple rounds of polyploidy and genome hybridization makes the identification of orthologous relationships between genes difficult. The genomes of three diploid species, *B*. *rapa* (AA, 2n = 20), *B*. *nigra* (BB, 2n = 16), and *B*. *oleracea* (CC, 2n = 18), have triplicated homologus counterparts of corresponding segments in the *Arabidopsis* genome as a result of whole-genome triplication, which occurred approximately 12 to 17 million years ago [8,32]. In addition, *B*. *oleracea* and *A*. *thaliana* diverged 15 to 20 million years ago [33].

**Table 2:** Datasets used to analyze the applicability and orthology of SNP markers based on the *Brassica rapa* genome to the *Brassica oleracea* genome.

Sequence	*Brassica rapa*	*Brassica oleracea*	*Arabidopsis thaliana*
EST	194,305	59,946	
Genome	198,585 (GSS^a^)	680,894 (GSS^a^)	Chromosome (5)
mRNA	3,965	1,772	-
Protein	2,062	1,735	32,615
nrProtein^b^	55,420	20,632	32,615

a. GSS: Genome Survey Sequence.

b. nrProtein: Final protein sequence as a non-redundant protein dataset for ortholog analysis

The unigenes of 7,645 SNP markers of *B*. *rapa*, which are related to disease resistance, were mapped to the genomes of *B*. *oleracea* and *A*. *thaliana* using BLAST (version 2.2.24) algorithm with an e-value of 1e-4 (Table 3). Among 20,632 unigenes of *B*. *oleracea* and 32,615 unigenes of *A*. *thaliana*, 3,914 (18.9%) and 13,506 (41.4%) were orthologous with genes (or protein) specified by the *B*. *rapa* SNP markers. In total, 8,518 (41.2%) genes for *B*. *oleracea* and 1,946 (5.9%) genes for *A*. *thaliana* were unique, respectively. Of 7,645 SNP markers, 425 were applicable to *B*. *oleracea*, and 142 were applicable to *A*. *thaliana* (Table 4). The *Brassica* and *Arabidopsis* genomes share about 87% sequence identity in their coding regions [20]. This feature has been extensively exploited and has resulted in a large number of comparative mapping studies between *Brassica* crops and *Arabidopsis* [22]. Li et al. [4] tested sequence-tagged markers from *B*. *rapa* for homology with the genomic sequence of *A*. *thaliana*. They found that 223 markers had homologs in the genome of *A*. *thaliana*, and that these were distributed throughout the genome, except for one homolog, which was located on the short arm of chromosome 2. *Brassica rapa* is diploidy and has a small genome size (529 Mb) compared with its close diploid relative *B*. *oleracea* (696 Mb) [6]. These characteristics are useful for the study of genomic traits. Previous comparative mapping studies of *Brassica* and *Arabidopsis* using molecular markers revealed extensive synteny between *B*. *oleracea* and *A*. *thaliana*, suggesting that knowledge gained in one species can be productively applied to the other [34,35].

**Table 3:** Orthologous and unique genes between *Brassica oleracea* and SNP markers based on the *Brassica rapa* genome.

	*Brassica rapa*	*Brassica oleracea*	*Arabidopsis thaliana*
nrProtein^a^ (%)	55,420 (100)	20,632 (100)	32,615 (100)
Orthologous genes (%)	17,810 (32.1)	3,914 (18.9)	13,506 (41.4)
Unique genes (%)	7,165 (12.9)	8,518 (41.2)	1,946 (5.9)

a. nrProtein: Final protein sequence as a non-redundant protein dataset for ortholog analysis

**Table 4:** The applicability of SNP markers based on the *Brassica rapa* genome to *Brassica oleracea*.

Organism	*Brassica oleracea*	*Arabidopsis thaliana*
Template number	742,612	5
Number of successful PCR markers	425	142
PCR success rate (%)	5.56	1.86

### Functional annotation and GO

**Fig 1:**
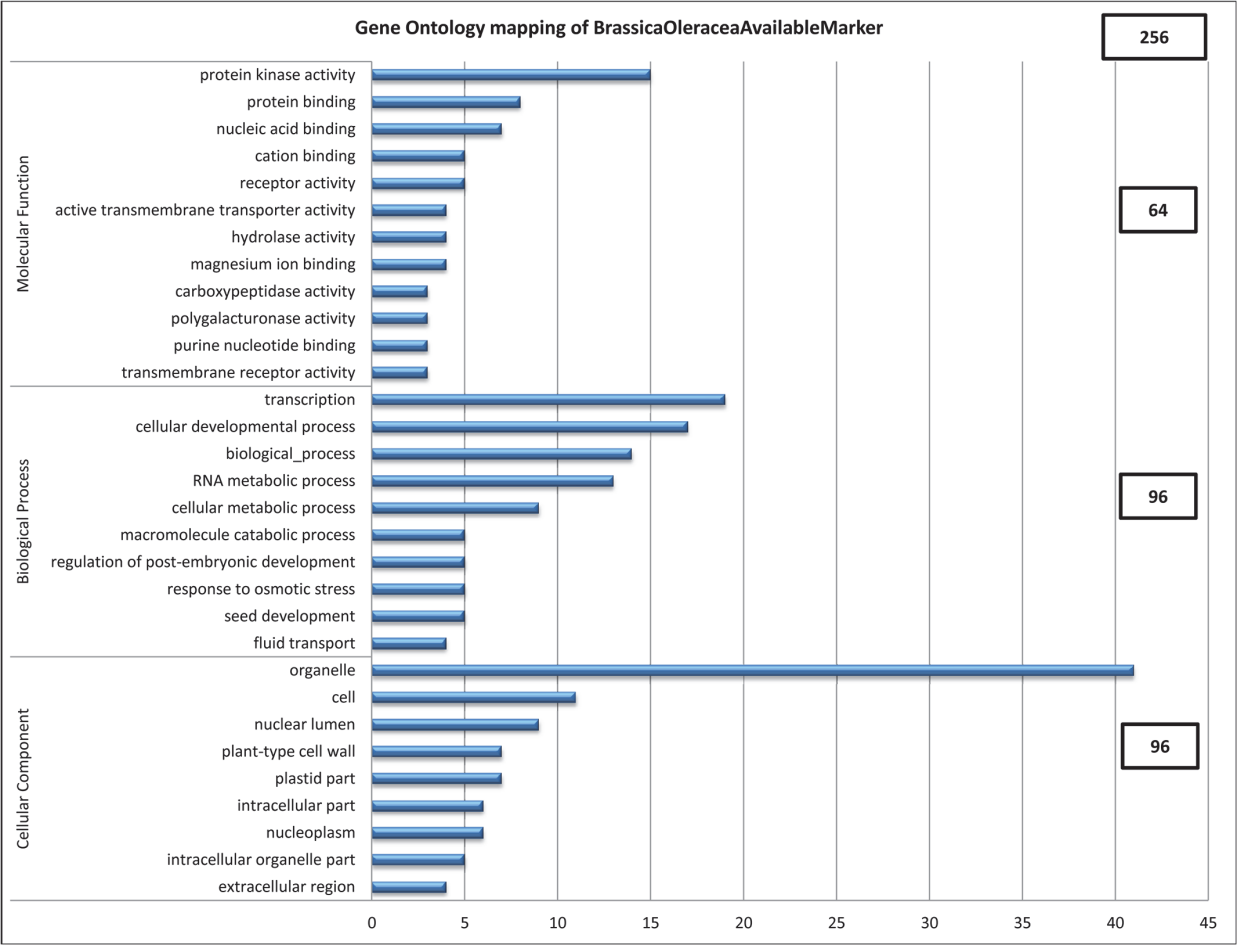
Gene Ontology (GO) mapping of cabbage genes using the sequence of the *Brassica oleracea* genome and 425 BRP based on the *Brassica rapa* genome.

A total of 6,412 of 6,856 genes, which were mapped to 7,645 SNP markers, were annotated with the reference genome data of *B*. *rapa* using functional annotation analysis (data not shown). Of 7,645 SNP markers, 425 BRP that were applicable to 256 genes in *B*. *oleracea*, were mapped with the genes of *B*. *oleracea* (Fig. 1). These genes were classified into three functional groups: molecular function (64 genes), biological process (96 genes), and cellular component (96 genes). Molecular function was subdivided into 12 categories: protein kinase activity (15), protein binding (8), nucleic acid binding (7), cation binding (5), receptor activity (5), active transmembrane transporter activity (4), hydrolase activity (4), magnesium ion binding (4), carboxypeptidase activity (3), polygalacturonase (3), purine nucleotide binding (3), and transmembrane receptor activity (3). Biological process was subdivided as follows: transcription (19), cellular developmental process (17), biological process (14), RNA metabolic process (13), cellular metabolic process (9), macromolecule catabolic process (5), regulation of post-embryonic development (5), response to osmotic stress (5), seed development (5), and fluid transport (4). Cellular component was subdivided into nine categories: organelle (41), cell (11), nuclear lumen (9), plant-type cell wall (7), plastid part (7), intracellular part (6), nucleoplasm (6), intracellular organelle part (5), and extracellular region (4).

**Fig 2:**
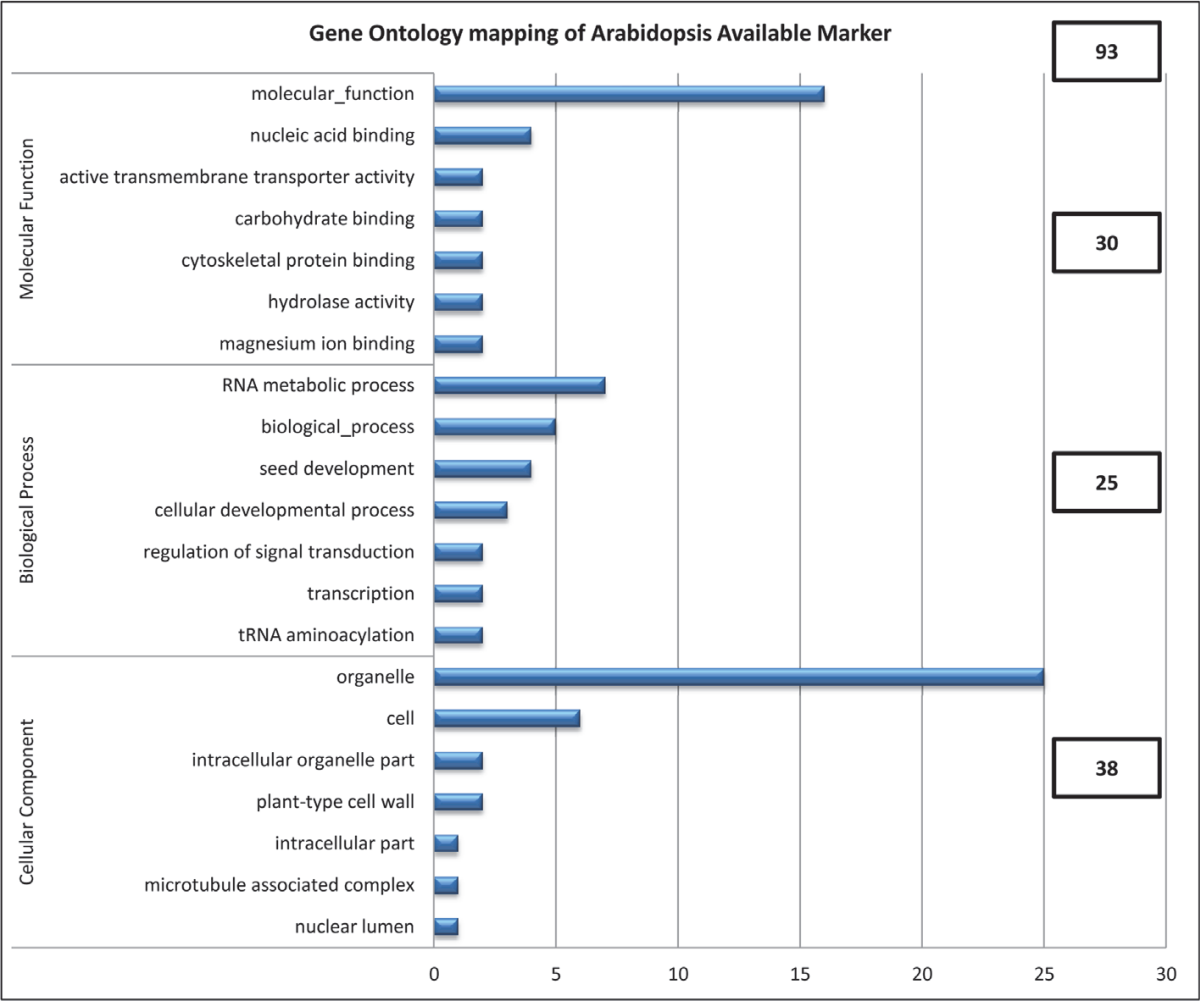
Gene Ontology (GO) mapping of *Arabidopsis thaliana* genes and the sequence of 142 SNP markers from *A*. *thaliana* based on the *B*. *rapa* genome.

The 142 SNPs that can be applied to 93 *A*. *thaliana* genes, as a result of GO annotation between the SNP sequences of *B*. *rapa* and sequence of the *A*. *thaliana* genome, were mapped with the genes of *A*. *thaliana* (Fig. 2). These genes were clustered into three functional categories: molecular function (30 genes), biological process (25 genes), and cellular component (38 genes). Molecular function was subdivided into seven categories: molecular function (16), nucleic acid binding (4), active transmembrane transporter activity (2), carbohydrate binding (2), cytoskeletal protein binding (2), hydrolase activity (2), and magnesium ion binding (2). Biological process was subdivided into seven categories: RNA metabolic process (7), biological process (5), seed development (4), cellular developmental process (3), regulation of signal transduction (2), transcription (2), and tRNA aminoacylation (2). Cellular component was subdivided seven categories: organelle (25), cell (6), intracellular organelle part (2), plant-type cell wall (2), intracellular part (1), microtubule associated complex (1), and nuclear lumen (1). Comparative genomics is rapidly emerging as a powerful tool for genome analysis and annotation [36]. The course of evolution for functional regions such as exons and regulatory sequences tends to be more conserved than that for nonfunctional regions; thus, local sequence similarities have implications for biological functionality. Nucleotide sequence conservation between *B*. *oleracea* and *A*. *thaliana* has been reported to be in the range of 75–90% for exons, compared to ≤ 70% for introns and intergenic regions. Thus, the genome-scale comparison of *Arabidopsis* with *Brassica* at the sequence level provides an excellent opportunity for the applicability of this phylogenetic footprinting approach to the annotation of plant genomes [37].

### Application *B*. *rapa* SNP primers to *B*. *oleracea*

**Fig 3:**
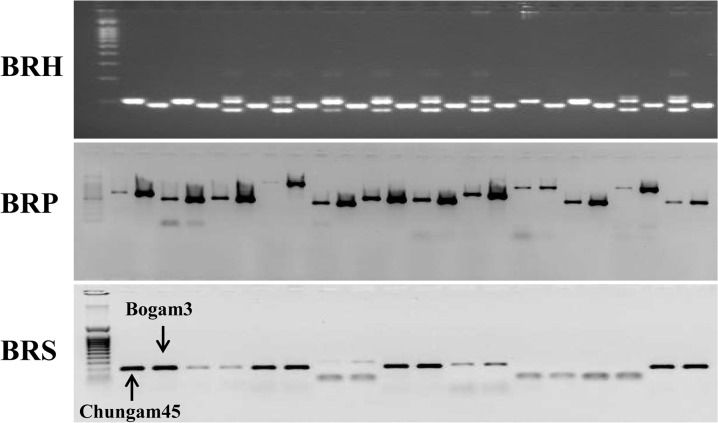
PCR amplification of genomic DNA from two cabbage varieties using SNP primers designed based on the *Brassica rapa* genome.

In this study, 693 SNP markers designed based on the *B*. *rapa* genome were tested for their applicability to *B*. *oleracea* using two cabbage varieties, Chungam45 and Bogam3 (Fig. 3 and Table 5). Of 145 BRH, 108 (74.5%) were amplified. In addition, 415 of 425 BRP (97.6%) were amplified, and 118 of 123 BRS (95.9%) were amplified using genomic DNA from two cabbage varieties. The amplification values for the BRP and BRS were higher than for the BRH. A total of 641 of 693 SNP markers from *B*. *rapa* were amplified using PCR, suggesting that these markers are beneficial molecular markers for *B*. *rapa* genetic analyses and breeding and that they can be applied to other *Brassica* species, including *B*. *oleracea*. These results provide valuable information that can be used for the utilization of *Brassica* in genomic studies and cabbage breeding.

**Table 5:** Amplification of SNP markers based on the *Brassica rapa* genome to *Brassica oleracea*.

Primer	No.	PCR amplification		
		Amplification	No amplification	Rate (%)
BRH^a^	145	108	37	74.5
BRP^b^	425	415	10	97.6
BRS^c^	123	118	5	95.9

a. BRH: SNP marker developed from the *Brassica rapa* genome for high-resolution melt (HRM) analysis.

b. BRP: SNP marker based on the *B*. *rapa* genome that could be applied to *B*. *oleracea*.

c. BRS: New SNP marker derived from a BRP designed for HRM analysis.
